# Breast cancer after polyacrylamide hydrogel augmentation: A clinicopathological analysis of 5 cases and literature review

**DOI:** 10.1016/j.radcr.2026.07.036

**Published:** 2026-08-05

**Authors:** Xiaomei Ma, Zhi Zhu

**Affiliations:** Department of Pathology, The Second Affiliated Hospital of Naval Military Medical University, Shanghai, China

**Keywords:** Breast augmentation by injection, Polyacrylamide hydrogel, Breast cancer, Imaging evaluation, Pathology

## Abstract

Polyacrylamide hydrogel (PAAG) injection was once widely used for cosmetic breast augmentation in China but was later banned due to severe long-term complications; PAAG deposition can interfere with breast imaging and delay the diagnosis of secondary breast cancer, bringing great clinical challenges. This study retrospectively analyzed 5 female patients diagnosed with breast cancer long after PAAG injection breast augmentation, summarized their clinical data, multimodality imaging manifestations, pathological features and long-term follow-up outcomes, and combined relevant published literature to further characterize this rare patient population. Our results demonstrated that breast cancer arising in PAAG-augmented breasts presents younger onset age, prominent imaging obscuration, high axillary lymph node metastasis rate and poor overall prognosis compared with conventional breast cancer. Routine breast ultrasound is recommended for long-term surveillance of PAAG recipients, and contrast-enhanced magnetic resonance imaging is the preferred examination for evaluating suspicious breast masses; standardized follow-up protocols and active pathological biopsy are essential for early diagnosis and timely intervention.

**Key Messages**.

Imaging plays a critical role in the early detection of breast cancer in patients with prior polyacrylamide hydrogel (PAAG) breast augmentation. A unique imaging obscuration phenomenon caused by retained PAAG differs from the findings in patients with other types of breast augmentation. Retained injectable material and related complications reduce the diagnostic sensitivity of mammography. Therefore, ultrasound is the first-line modality for routine follow-up, and MRI is reserved for further characterization of suspicious breast malignant lesions.

Alt-text: Unlabelled box dummy alt text

## Introduction

Among various postoperative complications of cosmetic breast augmentation, secondary breast cancer has become one of the most concerning issues for clinicians and patients [1]. Polyacrylamide hydrogel (PAAG) was popular for breast augmentation because of its non-toxic property, nonabsorbable characteristic and minimally invasive procedure without general anesthesia. However, its clinical use was officially banned in China in 2006 after around 10 years of clinical application, owing to a large number of reported adverse reactions and long-term complications [2]. Currently, clinical studies on PAAG-related breast lesions remain limited, and only 8 cases of breast cancer following PAAG injection augmentation have been documented in existing literature [[3], [4], [5], [6], [7]]. Although imaging is indispensable for early diagnosis of breast cancer in this special group, relevant systematic summaries are still insufficient. The complicated tissue changes induced by retained PAAG often lead to difficult imaging interpretation and delayed cancer diagnosis. In the present study, we systematically analyzed the clinical, imaging and pathological features of 5 new cases of breast cancer after PAAG breast augmentation, together with 8 previously reported cases from literature. This work aims to provide evidence for early diagnosis and lay a foundation for further related basic research.

## Case report

### Case 1

A 52-year-old female patient received bilateral PAAG injection for breast augmentation 14 years prior to presentation. Four years after the injection, she received PAAG removal and simultaneous breast reconstruction with silicone prostheses due to persistent breast discomfort. The patient received annual physical examinations thereafter with no abnormal findings. She had 3 children and a history of breastfeeding before augmentation, with no personal or family history of breast diseases.

### Imaging findings

**Fig. 1:**
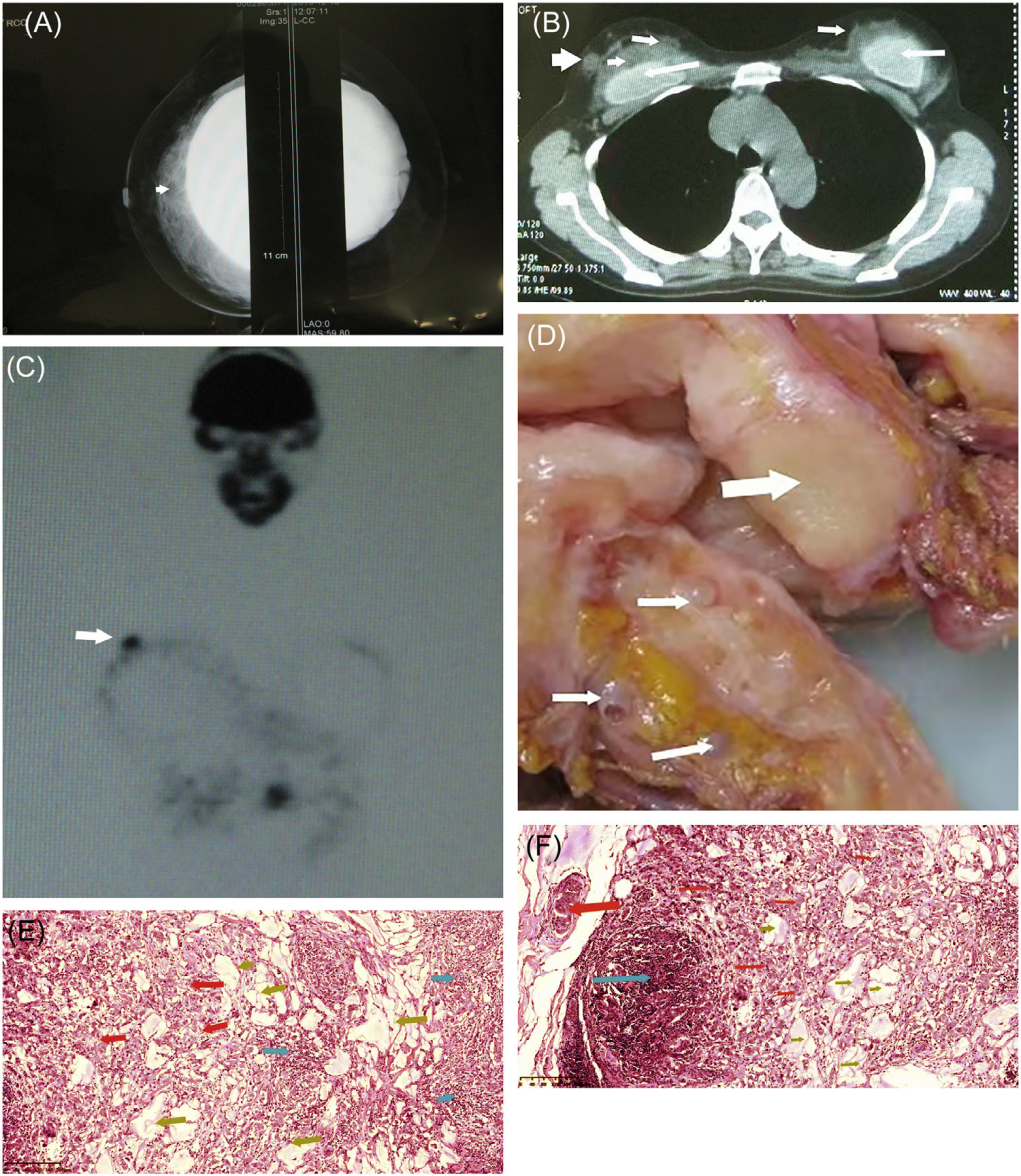
(A) Mammography of Case 1 shows bilateral silicone breast prostheses with compressed native breast tissue. Diffuse high-density retained injectable material extensively obscures the breast parenchyma (arrow), making it difficult to identify space-occupying lesions. (B) CT shows bilateral breast prostheses (left arrow) and a 2.2 × 2.0 × 1.0 cm malignant-appearing nodule in the right upper outer quadrant (thick right arrow). Multiple bilateral breast cysts are also seen (thin right arrow). (C) Whole-body PET-CT shows increased FDG uptake in the right upper outer breast nodule, consistent with breast cancer (arrow). No abnormal hypermetabolic lesions suggestive of distant metastasis are detected. (D) Gross specimen shows an infiltrative solid breast tumor with ill-defined borders (thick arrow). Numerous variable-sized cystic lesions filled with fluid are diffusely distributed in the breast tissue (thin arrows). (E) Histopathological image: Cancer cells grow in an infiltrative nested pattern with large cellular size (red arrow). A large number of inflammatory cells infiltrate the tumor stroma (blue arrow). Abundant basophilic mucinous foreign material (PAAG) is scattered in the tissue (green arrows) (HE staining, ×10). (F) Histopathological image: Metastatic tumor cells (thin red arrow) and mucinous foreign material (green arrow) are seen in the axillary lymph node. Mucinous material and tumor thrombus are present in the lymph node capsule (thick red arrow) (HE staining, ×10).

A solid breast mass was identified on routine breast screening. Mammography failed to clearly delineate the lesion (Fig 1A). Chest computed tomography (CT) revealed multiple cystic lesions in bilateral breasts with silicone prostheses in place (Fig 1B). A 2.2 × 2.0 × 1.0 cm nodule was detected in the upper outer quadrant of the right breast, highly suspicious for malignancy. Multiple right axillary lymph node metastases were also noted. Whole-body PET-CT excluded distant metastasis in other organs (Fig 1C).

### Management/follow up

Intraoperative tumor resection and frozen-section analysis were performed. Gross examination of the modified radical mastectomy specimen revealed numerous tiny cystic lesions measuring 0.1-0.5 cm (Fig 1D). Frozen-section diagnosis confirmed invasive ductal carcinoma, and the patient underwent right breast modified radical mastectomy. Paraffin-embedded sections showed tumor cells arranged in nests and sheets with significant cellular atypia; the lesion was classified as WHO grade 2 invasive ductal carcinoma (Fig 1E). Metastatic carcinoma was identified in 8 out of 27 resected axillary lymph nodes (8/27) (Fig 1F). Immunohistochemical results: Ki-67 (30%), ER (90% positive), PR (5% positive), HER2 (2+). FISH test confirmed HER2 gene amplification.

The patient received standard adjuvant chemotherapy after surgery. No recurrence or distant metastasis was observed during the 12-year follow-up period.

### Case 2

A 48-year-old female patient had a 14-year history of bilateral PAAG injection breast augmentation. A breast mass was detected in the right upper outer quadrant during routine ultrasound screening. She gave birth to 1 child and breastfed before augmentation, with no personal or family history of breast disorders.

### Imaging findings

**Fig. 2:**
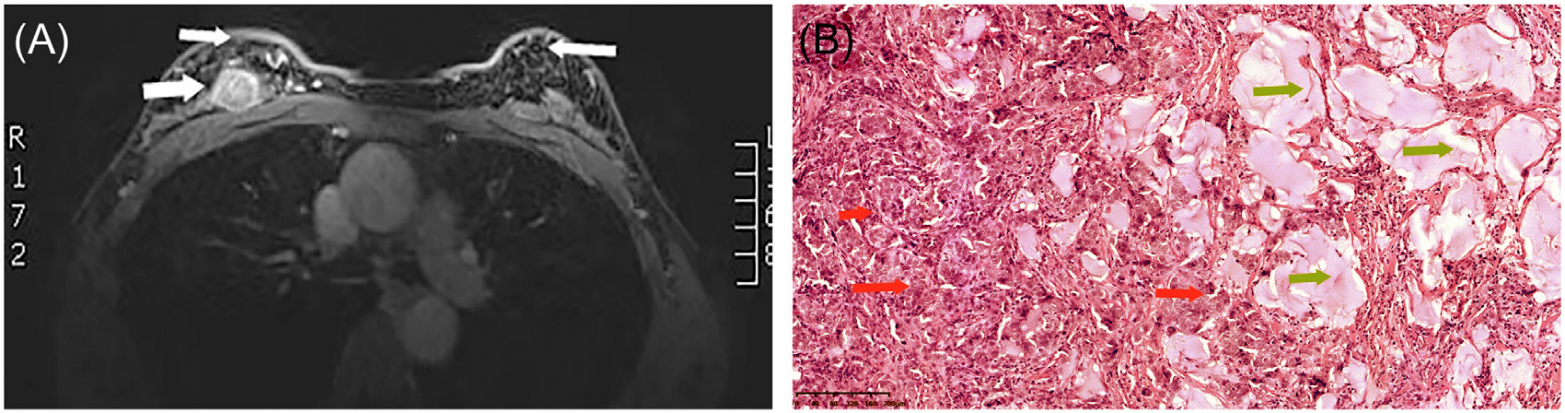
(A) Fat-saturated T2-weighted MRI shows a solid mass in the right breast (thick arrow) and diffuse cystic lesions in bilateral breasts (thin arrow). (B) Histopathology: tumor cells proliferate in sheets with marked cellular atypia (red arrow). A large amount of mucinous foreign material (PAAG) is distributed in the breast stroma (green arrow) (HE staining, ×10).

Magnetic resonance imaging (MRI) demonstrated a right breast nodule with markedly elevated FDG uptake, consistent with breast cancer; bilateral breast hyperplasia was also noted (Fig. 2A).

### Management/follow up

Preoperative core needle biopsy confirmed invasive breast carcinoma of the right breast and atypical ductal hyperplasia of the left breast. The patient underwent bilateral total mastectomy and right axillary lymph node dissection upon her request. All breast specimens presented a mucinous texture with diffuse transparent colloidal globules and viscous contents. A 1.5 × 1.3 × 1.0 cm tumor nodule was found in the right breast. Pathological diagnosis: WHO grade 3 invasive ductal carcinoma (Fig. 2B); no metastatic carcinoma in 26 resected axillary lymph nodes (0/26). Variable-sized cystic structures were diffusely present in the breast parenchyma. Immunohistochemistry: Ki-67 (40%), ER (90% positive), PR (80% positive), HER2 (0). A 0.6 × 0.6 × 0.3 cm lesion in the left breast was diagnosed as atypical ductal hyperplasia.

The patient received adjuvant radiotherapy and chemotherapy. No recurrence or metastasis was found during the 11-year follow-up.

### Case 3

A 34-year-old female patient underwent bilateral PAAG injection breast augmentation 9 years ago, and received PAAG removal surgery 2 years later due to breast discomfort. She presented with persistent dull pain in the right breast. The patient delivered a baby via cesarean section 9 months prior and had a 4-month breastfeeding history, with no personal or family history of breast disease.

### Imaging findings

Ultrasound identified a 1.0 cm right breast mass, initially suspected to be a fibroadenoma.

### Management/follow up

**Fig. 3:**
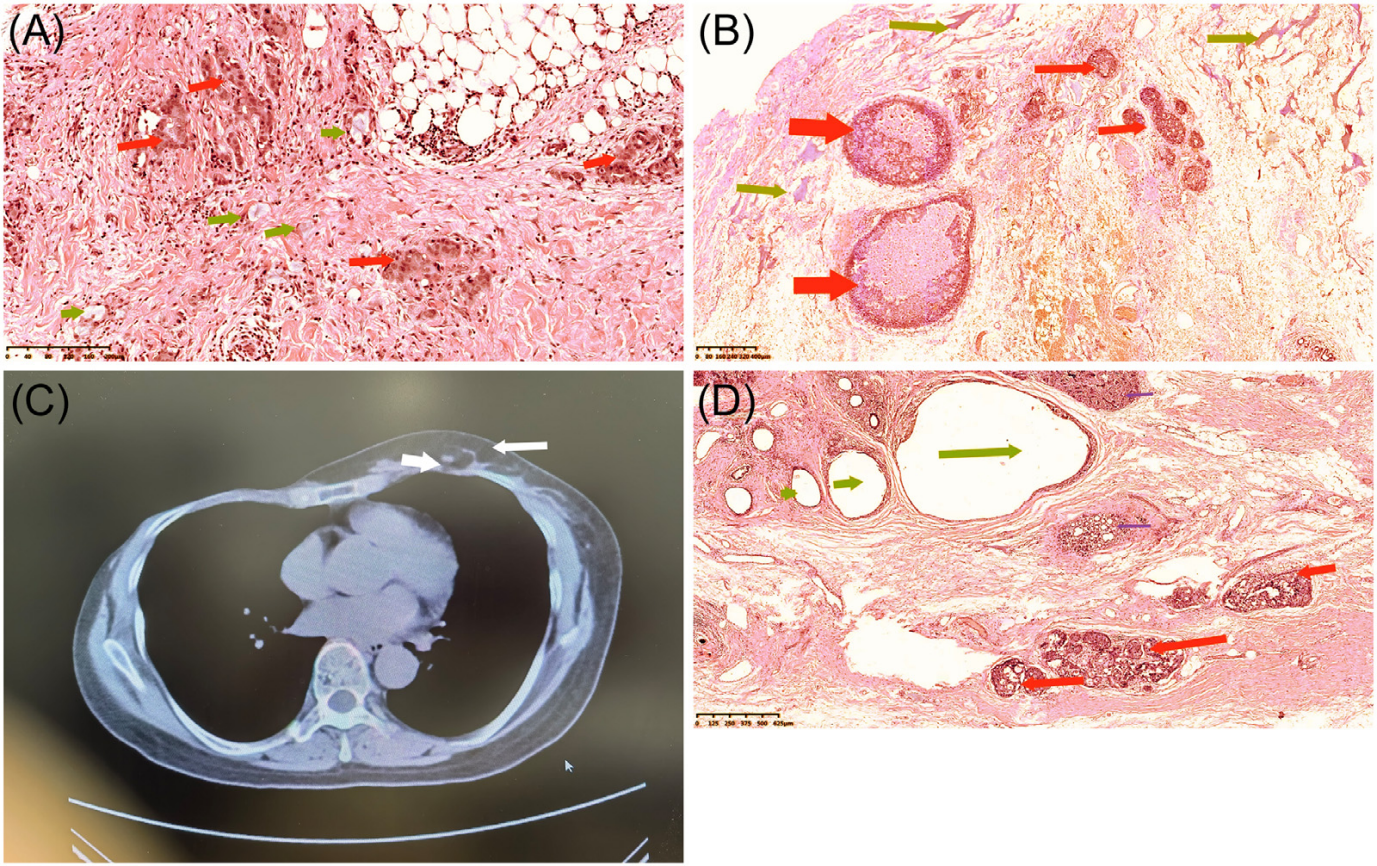
(A) Scattered invasive carcinoma cells are visible (red arrow). Polyacrylamide hydrogel (PAAG) is diffusely distributed without cyst formation (HE stain, ×10). (B) Atypical ductal epithelial hyperplasia is noted (thin red arrow). Foci of ductal carcinoma in situ with comedo necrosis are also present (thick red arrow). Diffuse PAAG deposition is seen within the breast stroma (green arrow) (HE stain, ×10). (C) Postoperative follow-up CT demonstrates complete resection of the right breast. Numerous cystic structures are observed in the left breast (left arrow), alongside a solid mass (right arrow). Two nodules measuring approximately 1.0 cm in maximum diameter are identified in the left breast, located near the nipple and upper outer quadrant respectively. The lesions are categorized as BI-RADS 3 breast masses. (D) Multiple cysts of varying sizes are scattered in the breast parenchyma (blue arrow, green arrow), with focal atypical epithelial hyperplasia (red arrow).

The patient received ultrasound-guided vacuum-assisted excision (Mammotome) of the breast lesion. Intraoperative frozen-section analysis revealed invasive ductal carcinoma, and she subsequently underwent right breast modified radical mastectomy. The breast specimen showed diffuse mucinous globules. Histopathological examination identified 2 components: high-grade ductal carcinoma in situ (approximately 60% of the lesion) and WHO grade 2 invasive ductal carcinoma (Fig. 3A and B), with comedo-type necrosis observed. Metastatic carcinoma was found in 3 out of 24 axillary lymph nodes (3/24), and PAAG material was detected within the lymph nodes. Immunohistochemistry: ER (70% positive), PR (70% positive), HER2 (2+), Ki-67 (20%). FISH excluded HER2 gene amplification. Adjuvant radiotherapy and chemotherapy were administered postoperatively.

Three months after surgery, follow-up imaging detected a new mass in the left breast (Fig. 3C), and biopsy confirmed atypical ductal hyperplasia (Fig. 3D). Lung metastasis developed 6 months after the operation, and the patient was subsequently lost to follow-up.

### Case 4

A 44-year-old female patient received bilateral PAAG injection breast augmentation 5 years ago. She accidentally palpated a painless hard mass in the right upper outer breast. She had 1 child and breastfed before augmentation, with no breast disease history or family predisposition.

### Imaging findings

**Fig. 4:**
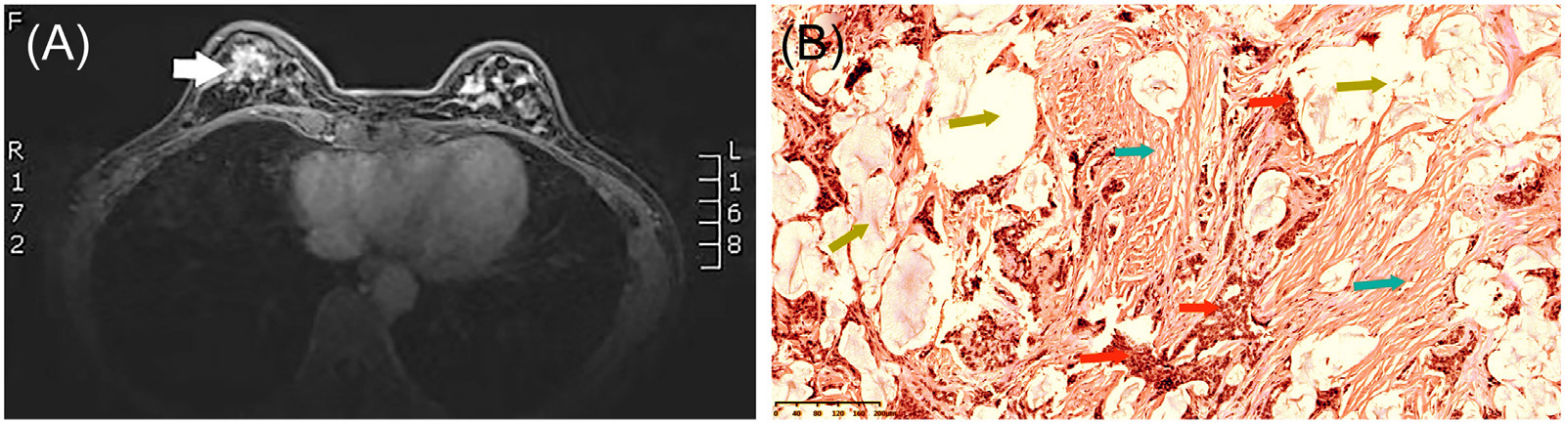
(A) MRI shows a solid mass in the right breast (arrow), with diffuse cystic lesions in both breasts. (B) Tumor cells are arranged in small nests (red arrow). Abundant collagen fibers are present in the stroma (blue arrows). Numerous PAAG microcapsules are scattered throughout the tissue (green arrow) (HE stain, ×10).

MRI revealed a 5.0 × 3.0 cm right breast mass, highly suggestive of breast cancer combined with axillary lymph node metastasis (Fig. 4A).

### Management/follow up

Core needle biopsy confirmed invasive breast carcinoma with right axillary lymph node metastasis. The patient received neoadjuvant chemotherapy, and the tumor shrank to 3.0 × 2.0 cm in size. Right breast modified radical mastectomy was then performed. Pathological diagnosis: WHO grade 2 invasive ductal carcinoma (Fig. 4B), with metastasis in 4 out of 9 axillary lymph nodes (4/9). Immunohistochemistry: ER (80% positive), PR (negative), HER2 (2+), Ki-67 (15%). FISH showed no HER2 amplification.

Adjuvant chemotherapy was given after surgery. Local recurrence occurred at 6 months, and lung metastasis developed at 9 months postoperatively.

### Case 5

A 37-year-old female patient had an 18-year history of bilateral PAAG injection breast augmentation. She underwent PAAG removal and simultaneous silicone prosthesis implantation 4 years after the initial injection due to discomfort. The patient self-palpated a right breast mass. She had two children and completed breastfeeding after augmentation, with no personal or family history of breast disease.

### Imaging findings

**Fig. 5:**
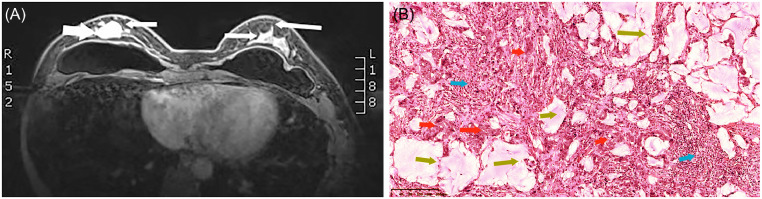
(A) Contrast-enhanced MRI of Case 5 demonstrates a solid mass in the right breast (thick arrow) with markedly increased FDG uptake, highly suggestive of breast cancer. Diffuse cystic lesions are visible bilaterally around the implanted silicone prostheses (thin arrow). (B) Tumor cells are arranged in cords and nests (red arrow), with lymphocytic infiltration in the stroma (blue arrow). Abundant basophilic mucinous foreign material consistent with PAAG is also noted (green arrow) (HE stain, ×10).

Contrast-enhanced MRI demonstrated a 1.5 × 1.0 cm right breast mass with intense enhancement, consistent with breast cancer. Bilateral breast fibrocystic changes were noted, and no axillary lymphadenopathy was detected (Fig. 5A).

### Management/follow up

Intraoperative frozen-section analysis diagnosed invasive ductal carcinoma for the right breast lesion and benign fibrocystic changes for the left breast. The patient underwent right breast modified radical mastectomy combined with bilateral silicone prosthesis removal. Pathological diagnosis: WHO grade 3 invasive ductal carcinoma (Fig. 5B), with metastasis in 3 out of 16 axillary lymph nodes (3/16). Immunohistochemistry: Ki-67 (40%), ER (80% positive), PR (70% positive), HER2 (1+). FISH ruled out HER2 amplification.

The patient remained free of recurrence within the first year after surgery but developed spinal metastasis at 12 months postoperatively; follow-up was lost thereafter.

## Discussion

### Etiology and demographics

Cosmetic breast augmentation is widely performed globally, with rapidly growing demand in China accompanied by increasing postoperative complications, including breast asymmetry, infection, gel migration, tissue deformation and palpable masses. PAAG was once popular in multiple Asian and Eastern European countries. However, frequent severe adverse complications led to its official clinical ban in China in 2006, while Western countries have never approved its clinical application. Currently, clinical studies focusing on PAAG-related breast lesions remain limited, with most reports concentrating on basic complications rather than secondary malignant tumors.

Palpable breast masses are the most common late complication of PAAG injection, mainly derived from gel aggregation, inflammatory granulomas or secondary neoplasms. Residual PAAG severely interferes with clinical palpation and imaging assessment, creating substantial difficulties in differentiating benign gel nodules from malignant tumors. Compared with silicone augmentation recipients, patients with prior PAAG injection face greater challenges in early breast cancer diagnosis. Clinicians often misattribute newly emerging breast lumps to chronic gel-related lesions, ultimately causing misdiagnosis and delayed treatment. Given the extremely limited reported cases of PAAG-associated breast malignancy, this study systematically analyzed 5 new clinical cases combined with 8 literature-reported Chinese cases to supplement relevant clinical evidence. All patients provided written informed consent for this retrospective analysis.

### Clinical and imaging findings

The pooled 13 cases (Table 1) indicated that PAAG-associated breast cancer predominantly affects young and middle-aged females. The mean age at PAAG injection was 33.2 years, and the mean age at cancer diagnosis was 37.8 years, significantly younger than patients with breast cancer after other augmentation procedures. The mean interval from injection to malignant diagnosis was 8.6 years, with a maximum latent period of 18 years, demonstrating a long disease incubation stage. A small number of cases developed malignancy within 1 year after injection, suggesting possible pre-existing occult breast lesions before augmentation.

**Table 1:** Clinicopathological features review of breast cancer following PAAG injection in the literature.

		Age	Breast augmentation years	Birth history	location	Tumor length (cm)	Diagnosis	Lymph node	ER/PR/Cerbb-2/fish	Follow up
Xiao et al. [3]	1	29	6 mo	NA	L	1.2	IDC	NON	NON	NA
	2	31	8 mo	NA	R	1.8	IDC	NON	NON	NA
Cheng et al. [4]	1	30	2 y	NA	R	6.0	IDC	14/25	+/+/0/N	2 y, metastasis
	2	44	5 y	Childless	L	7.0	IDC	1/18	−/−/0/N	1 y, metastasis
Zhao et al. [5]	1	43	10 y	1	L+R	R: 6.0 L: 0.8	IDC+ILC	L: 8/13 R: 11/11	+/+/0/N	2 mo, recur and metastasis
Chen et al. [6]	1	48	10 y	NA	L	10	IDC	Non	−/−/2+/N	2, no recur or metastasis
Yang et al. [7]	1	49	12 y	NA	L	5.0	IDC+DCIS	Non	Non	NA
	2	47	12 y	NA	L	0.2	Invasive carcinoma	Non	Non	NA
Our cases	1	52	14 y	3	R	2.3	IDC	8/27	±/2+/+	12 y, no recur or metastasis
	2	48	14 y	1	R	1.5	IDC	0/26	+/+/0/N	11 y, no recur or metastasis
	3	34	9 y	1^a^	R	1.0	IDC+DCIS	3/24	±/2±	6 mo, metastasis
	4	44	5 y	1	R	5.0	IDC	4/9	±/2±	6 mo, recur, 9 mo metastasis
	5	37	18 y	2^a^	R	1.5	IDC	3/16	±/1±	1 y, metastasis

DCIS, ductal carcinoma in situ; IDC, invasive ductal carcinoma; ILC, invasive lobular carcinoma; NA, not available.

a Birth after breast augmentation.

Diagnostic delay is the most prominent clinical feature of this disease. Diffuse nodularity and chronic tissue sclerosis caused by residual PAAG mask early malignant nodules, while large tumors are easily misdiagnosed as benign PAAG granulomas. Most patients are diagnosed at advanced tumor stages with a high axillary lymph node metastasis rate. Even patients with regular imaging follow-up exhibit frequent lymph node metastasis, which may be associated with local inflammatory suppression, weakened fibrous encapsulation of tumor cells, and accelerated lymphatic reflux that promotes tumor dissemination.

Mammography shows low sensitivity and specificity for PAAG-augmented breasts due to tissue masking effects. Ultrasound and MRI are the optimal imaging modalities for lesion evaluation. Routine ultrasound screening is strongly recommended for postoperative follow-up, while contrast-enhanced MRI is essential for further characterization of ultrasound-suspicious masses. A unique imaging obscuration phenomenon specifically exists in PAAG-augmented breasts: diffuse amorphous PAAG mixes with native breast tissue, forming abnormal background signals on imaging and completely masking small tumor nodules, which is the primary cause of clinical missed diagnosis [8].

### Pathological findings

Gross pathological examination of PAAG-injected breast tissue reveals extensive translucent cystic changes with granular, tough texture. Tumor lesions present ill-defined borders and are frequently mixed with foreign body granulomas and fibro-sclerotic tissue. Invasive ductal carcinoma is the dominant pathological type, consistent with conventional breast cancer. No significant differences in ER/PR and HER2 molecular subtypes were identified between this cohort and ordinary breast cancer patients.

Theoretically, diffuse PAAG deposition may induce multicentric or bilateral breast cancer, yet only one bilateral case was identified in the 13 enrolled cases, with no multifocal lesions observed. The tumor microenvironment is complicated by chronic inflammation, foreign body granulomas and extensive fibrosis. Seven of the 13 patients presented axillary lymph node metastasis with a higher metastatic burden than conventional breast cancer, largely attributable to diagnostic delay. Notably, 6 of 9 followed patients developed early distant metastasis within 2 years postoperatively, indicating a high early recurrence rate and poor prognosis.

### Treatment and literature discussion

The standard treatment for PAAG-associated breast cancer is modified radical mastectomy combined with complete residual PAAG removal, with immediate breast reconstruction not recommended. Routine ipsilateral axillary lymph node dissection and active pathological biopsy for all suspicious solid lesions are necessary to avoid missed diagnosis. Although cosmetic breast augmentation does not increase the overall incidence of primary breast cancer according to mainstream studies, PAAG injection may worsen patient prognosis.

To date, no direct evidence confirms the carcinogenic effect of PAAG. Existing studies verify that PAAG only induces persistent chronic inflammation and foreign body reactions in soft tissue, which may form a protumor microenvironment and accelerate the progression of pre-existing occult breast lesions. Consistent with previous findings, this cohort showed early postoperative metastasis and poor prognosis. Further research is required to explore the underlying mechanism between PAAG-induced chronic inflammatory microenvironment and breast cancer progression, and to establish standardized follow-up and diagnostic protocols for this special patient population.

### Conclusion

Breast cancer developing after PAAG injection breast augmentation has distinct clinical, imaging and pathological features compared with conventional breast cancer: younger onset age, commonly delayed diagnosis, difficult imaging evaluation, severe background interference on pathological slides, high axillary lymph node metastasis rate and unfavorable prognosis. Given that aggregated PAAG masks tumor lesions, delays patients’ medical consultation and interferes with clinicians’ judgment, breast ultrasound must be designated as the standard long-term surveillance modality for all individuals with prior PAAG injection. Active pathological biopsy is strongly recommended for all imaging-suspicious solid breast masses. A full understanding of these unique characteristics will facilitate timely diagnosis and standardized treatment for this special patient group. We call for more multicenter case accumulation in the future. Our team will further focus on establishing standardized imaging follow-up protocols and exploring the potential biological correlation between PAAG-induced chronic inflammatory microenvironment and the occurrence and progression of breast cancer.

### Imaging features

This study enrolled 5 patients with PAAG injection-related breast cancer, aged 37-52 years, with a postinjection interval of 5-18 years. Two patients underwent PAAG removal combined with silicone prosthesis implantation 4 years after augmentation due to adverse reactions. Three cases were detected by routine ultrasound screening, while the other 2 presented with self-palpated breast masses, with tumor maximum diameters ranging from 1.0 cm to 5.0 cm. All patients had no personal or family history of breast disease, with inconsistent childbearing timelines before and after augmentation.

Mammography showed poor diagnostic performance due to severe tissue masking by diffuse PAAG deposition. CT only visualized large tumors, whereas MRI achieved accurate diagnosis in 4 cases, with only one small lesion misdiagnosed as fibroadenoma. One case was complicated with bilateral breast hyperplasia. All patients received modified radical mastectomy, and 1 patient underwent additional contralateral total mastectomy due to atypical ductal hyperplasia.

### Histopathologic features

Gross specimens showed diffuse mucinous changes and scattered mucinous globules in breast tissues. Microscopically, residual PAAG existed as diffuse deposits or aggregated micro-globules. The dominant pathological type was invasive ductal carcinoma, accompanied by interstitial PAAG deposition and chronic inflammatory infiltration. One case contained 60% high-grade ductal carcinoma in situ, and another presented with bilateral fibrocystic breast changes combined with unilateral invasive ductal carcinoma. All 5 cases had axillary lymph node metastasis with varying positive burdens, and immunohistochemical molecular profiles differed individually.

Postoperative follow-up revealed distinct prognostic differences. 2 patients achieved long-term recurrence-free survival for 11 and 13 years, though one developed benign ductal atypical hyperplasia nodules 3 years after surgery. The remaining 3 cases experienced early adverse events, including postoperative metastasis within 6 months, local recurrence followed by distant metastasis within 9 months, and 1-year postoperative metastasis, respectively, suggesting poor short-term prognosis in high-risk patients.

## Clinical management and discussion

Combined with current cases and published literature, PAAG-associated breast cancer exhibits unique clinicopathological characteristics. The patients had a young mean onset age of 37.8 years, with an average 8.6-year latent period from injection to cancer diagnosis. This disease is characterized by prevalent diagnostic delay, advanced tumor stage, complicated imaging evaluation, severely disturbed pathological background, and high axillary lymph node metastasis rate. Such manifestations may result from blunted local inflammatory responses, weakened fibrous tumor confinement, and hyperactive lymphatic reflux that facilitates tumor metastasis.

No significant differences were found in histopathological manifestations and molecular subtypes between PAAG-related and conventional breast cancers. Nevertheless, PAAG injection causes greater diagnostic difficulties than silicone augmentation. Aggregated gel masks breast lesions, interferes with radiological and physical examination, and delays patient consultation, greatly reducing mammographic sensitivity. Therefore, ultrasound is recommended as the routine follow-up screening method, and MRI serves as the definitive diagnostic modality for suspicious malignant lesions. Consistent with this principle, the 2 regularly screened cases obtained favorable long-term prognosis.

Standard treatment includes modified radical mastectomy, complete residual PAAG clearance, and ipsilateral axillary lymph node dissection, combined with postoperative chemoradiotherapy when necessary. Active pathological biopsy is essential for all imaging-suspicious solid masses to avoid missed diagnosis. To date, no direct evidence proves the oncogenic effect of PAAG. Instead, PAAG-induced persistent chronic inflammation and foreign body reactions may form a pro-tumor microenvironment to promote the progression of occult breast lesions. Inconsistent with the theoretical speculation of multicentric and bilateral tumorigenesis, few bilateral lesions were observed in pooled cases. Future research will focus on establishing standardized imaging follow-up protocols and exploring the biological mechanism between PAAG-related chronic inflammatory microenvironment and breast cancer progression.

## Ethical approval

This study complies with the ethical principles outlined in the Declaration of Helsinki.

## Patient consent

Written informed consent was obtained from all individual participants included in this study.
